# Clinical characteristics and management of patients with nonalcoholic steatohepatitis in a real-world setting: analysis of the Ipsos NASH therapy monitor database

**DOI:** 10.1186/s12876-023-02794-4

**Published:** 2023-05-19

**Authors:** Karishma Shelley, Amy Articolo, Rakesh Luthra, Michael Charlton

**Affiliations:** 1 Novo Nordisk Inc, 800 Scudders Mill Road, Plainsboro, NJ USA; 2 Transplant Institute, Center for Liver Diseases, University of Chicago Biological Sciences, Chicago, IL USA

**Keywords:** Nonalcoholic fatty liver disease, Demography, Liver cirrhosis, Database

## Abstract

**Background:**

Nonalcoholic steatohepatitis (NASH) is the more severe, inflammatory type of nonalcoholic fatty liver disease (NAFLD). NASH, a leading indication for liver transplantation, is growing in prevalence. The extent of liver fibrosis, ranging from fibrosis stage (FS) of none (F0) to cirrhosis (F4), is a strong predictor of health outcomes. There is little information on patient demographics and clinical characteristics by fibrosis stage and NASH treatment outside of academic medical centers.

**Methods:**

We conducted a cross-sectional observational study using Ipsos’ syndicated NASH Therapy Monitor database, consisting of medical chart audits provided by sampled NASH-treating physicians in the United States in 2016 (n = 174) and 2017 (n = 164). Data was collected online.

**Results:**

Of 2,366 patients reported on by participating physicians and included in the analysis, 68% had FS F0–F2, 21% had bridging fibrosis (F3), and 9% had cirrhosis (F4). Common comorbidities were type 2 diabetes (56%), hyperlipidemia (44%), hypertension (46%), and obesity (42%). Patients with more advanced fibrosis scores (F3-F4) had higher comorbidity rates than patients with F0-F2. Commonly used diagnostic tests included ultrasound (80%), liver biopsy (78%), AST/ALT ratio (43%), NAFLD fibrosis score (25%), transient elastography (23%), NAFLD liver fat score (22%), and Fatty Liver Index (19%). Most commonly prescribed medications were vitamin E (53%), statins (51%), metformin (47%), angiotensin converting enzyme inhibitors (28%), and beta blockers (22%). Medications were commonly prescribed for reasons other than their known effects.

**Conclusion:**

Physicians in this study, drawn from a spectrum of practice settings, relied on ultrasound and liver biopsy for diagnosis and vitamin E, statins, and metformin for pharmacological treatment of NASH. These findings imply poor adherence to guidelines in the diagnosis and management of NAFLD and NASH.

**Plain language summary:**

Nonalcoholic steatohepatitis (NASH) is a liver disease caused by excess fat in the liver which can lead to liver inflammation and scarring (fibrosis), ranging from stage F0 (no scarring) to F4 (advanced scarring). The stage of liver scarring can predict the likelihood of future health problems, including liver failure and liver cancer. However, we do not fully understand how patient characteristics may vary at different stages of liver scarring. We looked at medical information from physicians treating patients diagnosed with NASH to understand how patient characteristics might differ based on the severity of their liver scarring. The majority (68%) of patients were stage F0-F2, with 30% having advanced scarring (F3-F4). In addition to NASH, many patients also had type 2 diabetes, high cholesterol, high blood pressure, and obesity. Patients with more advanced scarring (F3-F4) were more likely to have these diseases than patients with less severe disease (F0-F2). Diagnosis of NASH by participating physicians was based on tests including imaging (ultrasound, CT scan, MRI), liver biopsy, blood tests, and whether patients had other conditions that would put them at risk for NASH. The medications that the doctors prescribed most often to their patients included vitamin E and drugs to treat high cholesterol, high blood pressure, or diabetes. Medications were frequently prescribed for reasons other than their known effects. By understanding how patient characteristics vary by stages of liver scarring and how NASH is currently managed may help guide the evaluation and treatment of NASH when NASH-specific therapies become available.

## Background

Nonalcoholic fatty liver disease (NAFLD) is a disease characterized by the accumulation of fat in the liver [1]. NAFLD typically develops due to chronic caloric excess, frequently with lack of exercise, in the absence of excessive alcohol consumption [2]. Nonalcoholic steatohepatitis (NASH) is the more severe form of NAFLD, characterized by liver inflammation and hepatocyte injury in addition to fat accumulation [1]. The prevalence of NAFLD in North America is estimated to be 25% [3]. The estimated prevalence of NASH among biopsied NAFLD patients is 61% [4]. Liver failure and liver cancer secondary to NASH is the most common indication for liver transplantation for women and second most common for men in the United States (US) [5].

The persistent inflammation seen in patients with NASH can lead to liver fibrosis (scarring of the liver) [6]. The severity of liver fibrosis varies widely between individuals with NASH and is the result of the net effect of a host of susceptibility and protective factors [7, 8]. The extent of fibrosis in the liver of a patient with NASH is a strong predictor of disease progression and health outcomes [9]. Liver fibrosis severity varies from stage F0 (no fibrosis) to F4 (cirrhosis) [2] and can be measured directly by liver biopsy or more recently, estimated by non-invasive clinical testing and imaging [2]. Because of the inherent discomfort, risk and expense associated with liver biopsies, only a small minority of patients with NAFLD undergo liver biopsy [10]. Although liver biopsy is considered to be the gold-standard for fibrosis measurement, biopsies are prone to sampling error, with only 1/50,000 of the liver tissue evaluated in a single transcutaneous or transvenous biopsy [11–13]. There is also substantial intra- and inter-observer variance in all aspects of histological assessment of NASH [14].

Most studies evaluating variability in liver fibrosis measurements from different techniques have occurred in the context of well-controlled clinical trials or consortia of academic centers, typically involving physicians with specialized training in hepatology. The majority of healthcare in the United States is provided outside of academic medical centers [15]. There is an incomplete understanding of how patient characteristics vary by fibrosis stage in patients with NAFLD and NASH and how NASH is managed in real-world settings that include physicians with and without specialist training in the care of patients with liver disease. A greater understanding of how patient characteristics vary by fibrosis staging could influence guidance on the evaluation and management of NASH when NASH-specific therapies become available. Identification and characterization of patients with NASH by fibrosis stage could inform treatment patterns that adapt to patient disease severity.

This study sought to evaluate the demographic and clinical characteristics of NASH patients by their fibrosis stage, determined by biopsy and by non-invasive means. We also aimed to understand how diagnostic testing and treatment regimens varied by patient fibrosis stage, by physician specialty, and by patient ethnicity in a real-world setting (i.e. outside of the context of a clinical trial, as defined by the Food and Drug Administration [16]) including understanding why physicians prescribe specific medications to patients with NASH.

## Methods

### Study population and design

This study was a non-interventional, cross-sectional database study utilizing the Ipsos syndicated NASH Therapy Monitor database. The Ipsos NASH Therapy Monitor contains data from retrospective medical chart audits completed by eligible physicians in the United States. Data is collected annually from physicians who extract patient demographics, disease status, comorbidities, testing, and treatment data on their most recent 5–10 NAFLD/NASH patients [17]. All methods were carried out in accordance with relevant guidelines and regulations. The clinical data of the patients was collected from the database, and all of the data were anonymized before we used it in this study. There were not any administrative permissions required to access the raw data used in our study.

Data in the database was collected online from 174 physicians in the United States from September to November 2016 and by 164 physicians from September to November 2017. Data was collected from 1,622 patient records in 2016 and 1,521 patient records in 2017, for a total of 3,143 records. Participating physicians included primary care physicians (PCPs), endocrinologists, hepatologists, and gastroenterologists. Physicians were randomly recruited from a large access panel and were required to manage at least 20 patients with NASH per month. The data provided was based on a sample of the de-identified NASH patients that they personally managed. Patients were required to have been assigned a clinical diagnosis of NASH, regardless of diagnostic method. Patient records were included in the database if they had a recorded assessment of fibrosis or recorded assessment at diagnosis. Fibrosis stage was recorded from a biopsy report or estimated by non-invasive testing (combination of clinical laboratory and imaging techniques including, but not limited to Fibrosis-4 Index [FIB-4] or vibration controlled transient elastography (VCTE, assessed by Fibroscan™; Echosens, Paris, France). Patients were excluded if they had a NAFLD fibrosis score below − 1.455 without a NASH diagnosis via a biopsy. See Table 1 for study sample attrition.

**Table 1:** Attrition of reported patients by sampled physician specialty

Total patient sample (2016 and 2017)	Patient medical records provided by all healthcare providers	Patient medical records provided by Primary Care Physicians	Patient medical records provided by Gastroenterologists	Patient medical records provided by Hepatologists	Patient medical records provided by Endocrinologists
**All^**	3,143	220	1,491	822	610
NAFLD fibrosis score less than -1.455	377	50	166	65	96
No NASH diagnosis confirmed by biopsy	201	8	101	25	67
Unknown completeness*	629	60	243	71	255
Final sample	**2,366**	**142**	**1,174**	**735**	**315**

^Data reflects patients excluded from analysis and may overlap among the three variables

*No fibrosis score at diagnosis AND no current fibrosis score

NASH, nonalcoholic steatohepatitis; NAFLD, nonalcoholic fatty liver disease

*Source: Ipsos NASH Therapy Monitor (NASH-treating physicians in US reporting on patients seen in consultation in 2016 [n = 174] and 2017 [n = 164]; data collected online). Data © Ipsos 2022, all rights reserved.*

### Study measures

This study measured current patient demographics including age, sex, race, body mass index (BMI), smoking status, insurance type, and employment status (at the time the medical chart records were collected). Clinical characteristics such as symptoms at diagnosis, fibrosis stage, and current comorbidities were also measured. Data was also collected regarding patient experiences with NASH diagnosis and treatment. Measures included the specialty of the diagnosing physician, types of diagnostic tests performed, and types of treatments administered. As part of the chart audit, physicians also recorded reasons for prescribing certain NASH-related treatments.

### Statistical analyses

Descriptive categorical statistics are presented as numbers and percentage of patients in each category. Patient fibrosis stage could be determined by biopsy or by combinations of clinical and non-invasive tests. All analysis was conducted in SAS 9.4 (SAS Institute Inc., Cary, North Carolina, USA).

## Results

### Patient demographics by fibrosis stage

After excluding patients with NAFLD and patients with NASH who had an unknown fibrosis score both at diagnosis and at the current assessment, there were 2,366 patients available for analysis. Patient demographic data, sorted by liver fibrosis stage, are shown in Table 2. Most patients were male (58%), and the largest proportion of patients (36%) had a liver fibrosis stage of F2. Fewer patients had liver fibrosis of stages F0 (8%), F1 (25%), F3 (21%), and F4 (9%). Patients with F3-F4 fibrosis had higher average BMIs than patients with F0-F2 fibrosis. Patients with F4 fibrosis had the highest mean age, were more likely to be White, and were more likely to have public health insurance when compared to patients with F0-F3 fibrosis. The proportion of Hispanic patients was similar across fibrosis stages. A lower proportion of patients with F4 fibrosis were Black/African American than patients with F0-F3 fibrosis (Table 2). Less than 1% of patients enrolled in the study were participants in a clinical trial.

**Table 2:** Demographics of reported patients by fibrosis stage, for patients with a current fibrosis score

	Total	F0	F1	F2	F3	F4	Unknown*
	(n = 2,366)	(n = 189)	(n = 576)	(n = 843)	(n = 486)	(n = 218)	(n = 54)
**Gender, n (%)**							
Female	1000 (42.3)	79 (41.8)	238 (41.3)	377 (44.7)	189 (38.9)	94 (43.1)	23 (42.6)
Male	1366 (57.7)	110 (58.2)	338 (58.7)	466 (55.3)	297 (61.1)	124 (56.9)	31 (57.4)
**Mean age, years (SD)**	51.1 (11.2)	48.0 (12.1)	49.7 (11.3)	50.9 (10.5)	51.0 (10.6)	58.3 (11.9)	53.2 (10.4)
**Mean BMI, kg/m** ^**2**^ **(SD)**	35.3 (8.6)	34.3 (7.1)	33.7 (7.7)	34.7 (8.3)	37.9 (8.2)	37.5 (12.4)	34.3 (4.4)
**Ethnicity, n (%)**							
White	1267 (53.6)	109 (57.7)	299 (51.9)	436 (51.7)	248 (51.0)	139 (63.8)	36 (66.7)
Black	526 (22.2)	41 (21.7)	118 (20.5)	197 (23.4)	134 (27.6)	31 (14.2)	5 (9.3)
Hispanic	470 (19.9)	33 (17.5)	120 (20.8)	175 (20.8)	91 (18.7)	39 (17.9)	12 (22.2)
Asian	82 (3.5)	5 (2.6)	25 (4.3)	31 (3.7)	11 (2.3)	9 (4.1)	1 (1.9)
Other	21 (0.9)	1 (0.5)	14 (2.4)	4 (0.5)	2 (0.4)	0 (0.0)	0 (0.0)
**Insurance type, n (%)**							
Private	1559 (65.9)	123 (65.1)	401 (69.6)	575 (68.2)	318 (65.4)	109 (50.0)	33 (61.1)
Public	718 (30.3)	52 (27.5)	153 (26.6)	244 (28.9)	149 (30.7)	101 (46.3)	19 (35.2)
Other	74 (3.1)	12 (6.3)	18 (3.1)	20 (2.4)	17 (3.5)	5 (2.3)	2 (3.7)
Uninsured	15 (0.6)	2 (1.1)	4 (0.7)	4 (0.5)	2 (0.4)	3 (1.4)	0 (0.0)
**Employment status, n (%)**							
Employed	1567 (66.2)	137 (72.5)	418 (72.6)	567 (67.3)	322 (66.3)	88 (40.4)	35 (64.8)
Disability leave	206 (8.7)	3 (1.6)	27 (4.7)	73 (8.7)	56 (11.5)	42 (19.3)	5 (9.3)
Not employed	244 (10.3)	25 (13.2)	53 (9.2)	99 (11.7)	45 (9.3)	19 (8.7)	3 (5.6)
Retired	262 (11.1)	17 (9.0)	42 (7.3)	81 (9.6)	49 (10.1)	65 (29.8)	8 (14.8)
Unknown	87 (3.7)	7 (3.7)	36 (6.3)	23 (2.7)	14 (2.9)	4 (1.8)	3 (5.6)
**Smoking status, n (%)**							
Current	574 (24.3)	46 (24.3)	135 (23.4)	216 (25.6)	120 (24.7)	47 (21.6)	10 (18.5)
Former	730 (30.9)	42 (22.2)	176 (30.6)	263 (31.2)	157 (32.3)	75 (34.4)	17 (31.5)
Never	954 (40.3)	86 (45.5)	232 (40.3)	328 (38.9)	195 (40.1)	90 (41.3)	23 (42.6)
Unknown	108 (4.6)	15 (7.9)	33 (5.7)	36 (4.3)	14 (2.9)	6 (2.8)	4 (7.4)

Abbreviations: BMI, body mass index; SD, standard deviation. Fibrosis stages range from none (F0) to cirrhosis (F4)

*Unknown at current appointment (when medical chart records were collected) but known at diagnosis

*Source: Ipsos NASH Therapy Monitor (NASH-treating physicians in US reporting on patients seen in consultation in 2016 [n = 174] and 2017 [n = 164]; data collected online). Data © Ipsos 2022, all rights reserved*

### Patient demographics by ethnicity

Just over half (54%) of patients included in the study were White. The next most common patient ethnicities were Black/African American (22%) and Hispanic (20%). There were relatively few patients of Asian descent (3%) and other ethnicities (< 1%). Ethnicity data was not available for ten patients. Mean age was similar across ethnicities (White, 52.1 years; Black, 50.0 years; Hispanic, 50.0 years; Asian, 51.0 years). The majority (64%) of White patients were male and 55% of Black patients were male; however, a majority of females were Hispanic (53%). White (73%) and Asian (73%) patients were most likely to have private health insurance. Black patients included in this study had the highest mean BMI (36.7 kg/m^2^), were most likely to be on public insurance (41%) and were most likely to have a history of smoking (66% were current or former smokers). Mean BMI (33.1 kg/m^2^) was lowest among Asian patients; these patients were also the least likely to have a history of smoking (59% had never smoked before). Between 61% and 69% of patients were currently employed with the rate of employment highest among White patients and lowest among Hispanic patients. Black patients had the highest rates of disability leave (14%) and White patients had the lowest rates of disability leave (7%).

### Patient symptoms and comorbidities by fibrosis stage

Patient symptoms reported at the time of diagnosis are reported in Table 3. Overall, the most commonly reported symptom at time of diagnosis was fatigue, followed by weight gain, and general weakness. Right upper quadrant pain was more common in patients with higher fibrosis scores. Patients with F0 fibrosis were less likely to report fatigue than patients with F1-F4 fibrosis. Overall, just over one-quarter of patients were asymptomatic at the time of diagnosis; being asymptomatic was most common among patients with F0 fibrosis and least common among patients with F4 fibrosis.

**Table 3:** Symptoms of reported patients at diagnosis by fibrosis stage, for patients with a current fibrosis score

Symptoms at diagnosis	Fatigue	Weight gain	Asymptomatic	General weakness	Hepatomegaly	Right upper quadrant pain	Sleep Apnea	Signs of liver disease	Weight loss	Other
Total (n = 2,312)	40%	37%	27%	25%	20%	18%	17%	9%	2%	2%
F0 (n = 189)	27%	33%	45%	10%	4%	5%	11%	2%	2%	2%
F1 (n = 576)	41%	35%	30%	25%	16%	13%	16%	5%	1%	2%
F2 (n = 843)	43%	37%	30%	28%	22%	17%	17%	4%	2%	1%
F3 (n = 486)	41%	43%	18%	28%	28%	24%	20%	8%	3%	3%
F4 (n = 218)	38%	30%	10%	25%	26%	28%	14%	44%	6%	6%

Fibrosis stages range from none (F0) to cirrhosis (F4). Patients with unknown fibrosis stage not shown (n = 54)

*Source: Ipsos NASH Therapy Monitor (NASH-treating physicians in US reporting on patients seen in consultation in 2016 [n = 174] and 2017 [n = 164]; data collected online). Data © Ipsos 2022, all rights reserved*

Patient comorbidities by fibrosis stage are reported in Table 4. Overall, the most common comorbidities among the patients with NASH were type 2 diabetes (T2D), hyperlipidemia, hypertension, and obesity. Broadly, patients with worse fibrosis scores (F3-F4) had higher rates of comorbidities than patients with better fibrosis scores (F0-F2). The prevalence of T2D, hyperlipidemia, obesity, cardiovascular disease, depression, hypothyroid, and chronic kidney disease (CKD) was generally higher among patients with F3-F4 fibrosis. The variation in prevalence of metabolic comorbidities was most notable for T2D and CKD, which increased with each increase in fibrosis stage.

**Table 4:** Comorbidities of reported patients by fibrosis stage, for patients with a current fibrosis score

Comorbidities	T2D	Hyperlipidemia	Hypertension	Obesity	Insulin resistance	Metabolic syndrome	CVD	Depression	Hypothyroid	CKD
Total (n = 2,312)	56%	50%	46%	42%	27%	17%	15%	11%	9%	8%
F0 (n = 189)	42%	33%	29%	32%	22%	12%	5%	9%	5%	1%
F1 (n = 576)	47%	39%	40%	30%	22%	15%	10%	10%	9%	3%
F2 (n = 843)	59%	55%	49%	42%	29%	18%	14%	11%	8%	6%
F3 (n = 486)	63%	58%	55%	56%	31%	19%	20%	13%	9%	14%
F4 (n = 218)	64%	52%	49%	56%	29%	22%	25%	21%	13%	22%

Abbreviations: T2D, type 2 diabetes; CVD, cardiovascular disease; CKD, chronic kidney disease. Fibrosis stages range from none (F0) to cirrhosis (F4). Patients with unknown fibrosis stage not shown (n = 54).

*Source: Ipsos NASH Therapy Monitor (NASH-treating physicians in US reporting on patients seen in consultation in 2016 [n = 174] and 2017 [n = 164]; data collected online). Data © Ipsos 2022, all rights reserved*

### Diagnostic testing

**Fig. 1:**
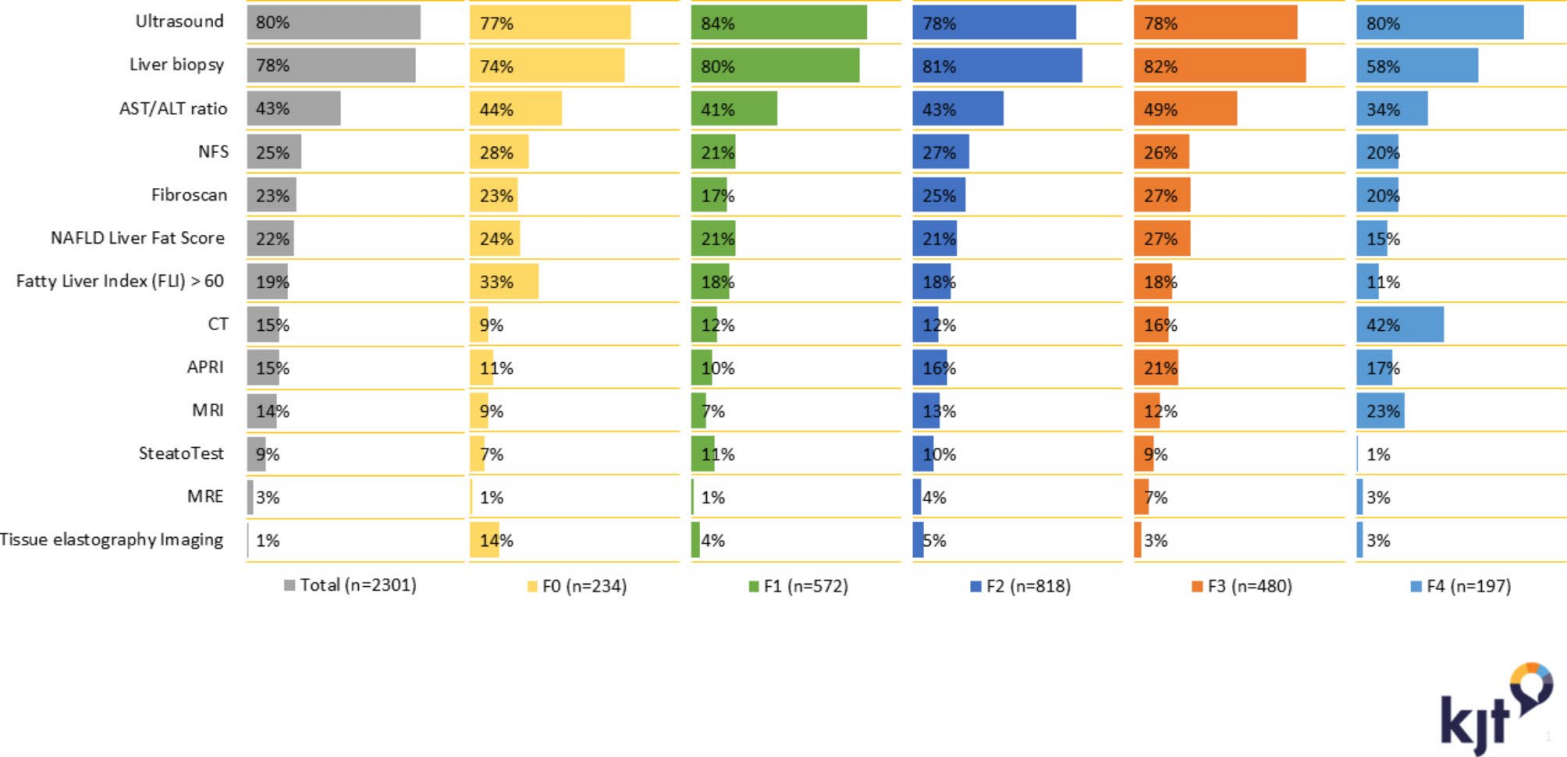
Tests performed at diagnosis by fibrosis stage (at diagnosis) of reported patients Abbreviations: AST/ALT, aspartate transaminase/alanine transaminase; APRI, AST to platelet ratio index; CT, computed tomography; MRE, magnetic resonance elastography; MRI, magnetic resonance imaging; NAFLD, nonalcoholic fatty liver disease; NFS, NAFLD fibrosis score. Patients with unknown fibrosis stage at diagnosis not shown (n = 65) *Source: Ipsos NASH Therapy Monitor (NASH-treating physicians in US reporting on patients seen in consultation in 2016 [n = 174] and 2017 [n = 164]; data collected online). Data © Ipsos 2022, all rights reserved*

Participating physicians used a variety of tests, including multiple screening or diagnostic approaches, to diagnose NAFLD/NASH and to assess severity of disease. The most commonly used diagnostic tests were ultrasound (80%), percutaneous liver biopsy (66%), AST/ALT ratio (43%), NAFLD fibrosis score (25%), transient elastography (23%), NAFLD liver fat score (22%), Fatty Liver Index (19%), computerized tomography (CT) (15%), AST/platelet ratio index (15%), magnetic resonance imaging (MRI) (14%), and transjugular liver biopsy (12%). When segmented by patient fibrosis stage at diagnosis, ultrasounds were performed most frequently in F1 patients (Fig. 1). MRIs and CT scans were performed at the highest rate in patients with F4 fibrosis. Magnetic resonance elastography (MRE) was performed at the highest rate in patients with F3 fibrosis. The Fatty Liver Index and tissue elastography imaging were used more often among patients with F0 than among patients with higher fibrosis stages.

**Fig. 2:**
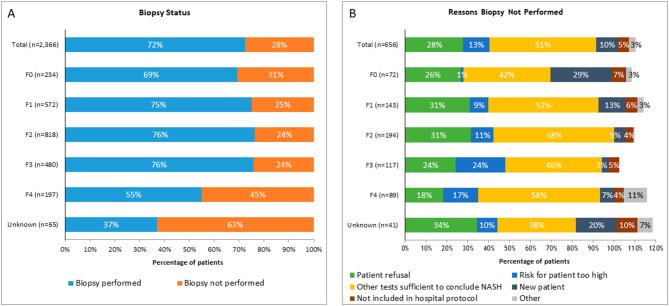
(**A**) Frequency of liver biopsy at diagnosis and (**B**) reasons for biopsy not being performed *Source: Ipsos NASH Therapy Monitor (NASH-treating physicians in US reporting on patients seen in consultation in 2016 [n = 174] and 2017 [n = 164]; data collected online). Data © Ipsos 2022, all rights reserved*

Overall, at diagnosis, liver biopsies were performed in 72% of patients in this study (Fig. 2A). Patients with F4 fibrosis had the lowest proportion (55%) of biopsies performed. Patients with F0 fibrosis had the second lowest proportion (69%) of biopsies performed, whereas 75–76% of patients with F1-F3 fibrosis had biopsies performed. When liver biopsies were not performed, physicians listed the reason the biopsy was not performed (Fig. 2B). The most common reason for lack of biopsy was ‘other tests sufficient to conclude NASH,’ followed by patient refusal.

Biopsy rates and reasons for lack of biopsy were also segmented by diagnosing physician specialty. Biopsy rates were broadly similar among PCPs, endocrinologists, gastroenterologists, and hepatologists (72-74% across all four diagnosing physician specialties). Rationale for not performing liver biopsy varied with specialty. Endocrinologists (56%) and gastroenterologists (55%) were more likely than PCPs (50%) and hepatologists (48%) to not perform a biopsy because other tests were deemed sufficient to conclude NASH. However, hepatologists, gastroenterologists, and PCPs were less likely to have biopsied patients with F4 fibrosis (69%, 56%, and 40% biopsied, respectively) than patients with F0-F3 (72–77%, 66–79%, and 62–80% biopsied, respectively). Endocrinologists diagnosed too few patients (n = 3) with F4 fibrosis to draw conclusions. Gastroenterologists biopsied a smaller proportion of patients (66%) with F0 fibrosis than did hepatologists (77%).

### Treatments for NASH

Overall, the most commonly prescribed medications for patients with NASH in this study were vitamin E (53%), statins (51%), metformin (47%), angiotensin converting enzyme (ACE) inhibitors (28%), and beta blockers (22%). Glucagon-like peptide-1 receptor agonists (GLP-1 RAs) (13%), Sodium-glucose co-transporter 2 (SGLT2) inhibitors (11%), pioglitazones (11%), dipeptidyl peptidase 4 (DPP-4) inhibitors (7%), and orlistat (9%) were prescribed in a minority of patients. Vitamin E use was highest in patients with F2 (58%) and F3 (55%) fibrosis. Patients with F0 (44%), F1 (48%), and F4 (40%) fibrosis had lower rates of vitamin E use. Pioglitazone prescription rates increased at higher patient fibrosis stages: from 8% of patients at F0 to 16% of patients at F4. In contrast, there were no obvious trends in prescriptions of GLP-1 RA/SGLT2/DPP4/orlistat when segmented by patient fibrosis stage. Beta blockers were prescribed at higher rates in patients with higher fibrosis stages: F0 (8%), F1 (17%), F2 (21%), F3 (25%), and F4 (45%), consistent with the higher prevalence of systemic hypertension with more advanced fibrosis stage in this study. Statins were prescribed at similar levels (between 40% and 60% of patients) across patient fibrosis stages with no clear trends. Of particular note is that the most common reasons for prescribing statins (from a predefined list) were to assist weight loss (54%) and to improve/reverse steatosis (42%). The top reason physicians said they prescribed orlistat (91%), SGLT2 inhibitors (70%), GLP-1 RAs (67%), pioglitazone (56%), DPP4 inhibitors (55%), metformin (46%), and vitamin E (40%) was to assist with weight loss; the second most common reason to prescribe these medications was to improve/reverse steatosis.

## Discussion

NASH is highly prevalent and is an increasingly common cause of cirrhosis, liver failure and liver cancer [2]. Liver fibrosis stage is the clinical parameter most closely linked with risk of negative health outcomes [9]. This analysis of a “real-world”, cross-sectional database study utilizing the Ipsos NASH Therapy Monitor database, including over 2,000 patients with NASH cared for by 170 physicians in multiple specialties, all of whom treat over 20 patients with NASH per month, has produced several important insights into how patients with NALFD and NASH may be being diagnosed and managed in non-academic settings. In interpreting the results of this analysis, it is important to consider that the provider participants in this study have a level of experience in the evaluation and management of NASH that would be most commonly encountered among providers at academic medical centers. This is important as only about 6% of hospitals in the United States are classified as academic medical centers [15]. The evaluation and management patterns reported by the contributing providers of the Ipsos NASH Therapy Monitor database, while reflecting “real world” practice in the sense that the study occurred outside of the context of clinical trials, may not be reflective of providers with less experience in the evaluation and management of NASH. Nonetheless, this analysis indicates challenges and opportunities in attenuating the health effects of the NASH epidemic.

There are several important aspects of this study and findings. The first is that this study included a large proportion of patients (> 70%) who received a liver biopsy. This is greater than reported in other real-world settings [18]. Among patients with NASH identified by ICD-10 coding in a large healthcare system, only ~ 1% had undergone liver biopsy [19]. In a cross-sectional survey of patients diagnosed with NASH, 53% reported having a liver biopsy to confirm their diagnosis [20]. Our analysis included patients relatively reliably ascertained as having NASH by the healthcare professionals participating in the chart review. The lowest frequency of liver biopsy was seen in patients assessed as having F4 fibrosis, for whom the most common reason physicians cited for not performing liver biopsies was that other tests were felt to be sufficient to conclude cirrhosis due to NASH. It is possible, for example, that morphological changes on imaging, e.g., nodularity on ultrasound, in the context of biochemical changes and features of the metabolic syndrome, were felt to be sufficient to make a diagnosis of NASH cirrhosis. In recent years, guidance from professional organizations (such as American Association for the Study of Liver Diseases [AASLD] and the American Gastroenterological Society) has advocated for increased use of non-invasive tests to rule out advanced fibrosis with non-invasive tests and only turning to liver biopsy when there is diagnostic doubt or for clinical trials [21, 22]. Additionally, the physicians who provided patient record data self-identified as treating a large number (≥ 20/month) of patients with NAFLD/NASH, and thus may be more comfortable with utilizing biopsy in the evaluation of patients with NASH. The high rate of biopsy in our study cannot be explained by participation in clinical trials because very few patients (< 1% of patients) included in this study participated in clinical trials. The observation that hepatologists and gastroenterologists performed fewer biopsies in patients with cirrhosis (F4 fibrosis) than in patients with F0-F3 fibrosis is of interest and may reflect more experience in interpreting non-invasive tests, including imaging. Despite the well documented variation in the prevalence of higher genotypes conveying greater risk of more advanced fibrosis with ethnicity e.g., for *HSD17B13* and *PNPLA3* [23, 24], ethnicity was not predictive of stage of fibrosis in this analysis. It is possible that genetic and environmental susceptibility and risk factors, e.g., variation in consumption of high-risk nutrients, offset. The observed lack of predictivity of ethnicity for more advanced liver disease in this study is in keeping with the lack of predictivity of ethnicity for end stage liver disease requiring liver transplantation in a national study of over 80,000 liver transplant recipients [25]. Interestingly, for the overall cohort of patients, between 61% and 69% were currently employed. However, there was a trend that patients with lower fibrosis scores (F0-F1) were associated with higher rates of employment (about 73%) than those with more advanced fibrosis (F4, employment rate of 40%). This may reflect the impact of NASH and related comorbidities on quality of life and the ability to complete necessary tasks to maintain employment. This may be an important area for future research.

Abdominal ultrasounds, which have some qualitative utility in detecting hepatic steatosis and ultrastructural changes of cirrhosis [26], were the most commonly used imaging modality, performed in over 70% of patients at each stage of fibrosis. Abdominal ultrasound is frequently used to evaluate abnormal liver biochemistries and to guide liver biopsy [27, 28]. Utilization of non-imaging-based methods to assess liver fibrosis was highly variable, without clear distinction in utilization patterns between eventual fibrosis stages or type of provider. The best performing blood test-based biomarker in this analysis, NAFLD fibrosis score, was used in approximately one-quarter of patients. Of the imaging techniques reported to be of utility in predicting risk for advanced liver disease, transient elastography (Fibroscan) was the most common, with MRE used rarely. A minority of patients who underwent a liver biopsy had one of the tests currently recommended in the NAFLD and NASH diagnosis and management guidance documents from the European Association for the Study of the Liver (EASL) and AASLD (NAFLD fibrosis score, transient elastography, or MRE) [28, 29]. These findings suggest a substantial potential benefit of dissemination and awareness of practice guidance.

Pharmacotherapy of NASH in this study was particularly striking. There are currently no Food and Drug Administration (FDA) approved treatments for NASH and there is limited evidence on what types of treatment are appropriate for patients with varying degrees of NASH severity or liver fibrosis. Because there are no FDA approved treatments specific to NASH, NASH management relies on general lifestyle improvements and treatment of comorbidities [22]. The most commonly prescribed treatments specifically to treat NASH in this study were vitamin E, statins, and metformin. Of these, only vitamin E has been shown to have any histological efficacy in randomized clinical trials [30, 31]. Pioglitazone, which demonstrated histological efficacy (reduction in lobular inflammation and steatosis) in the PIVENS trial but was not associated with improvement in fibrosis scores, [32] was more likely to be used in patients with more advanced fibrosis (8% of patients with F0 vs. 16% with F4). Use of GLP-1 RAs, SGLT2 inhibitors, and pioglitazone can be used to improve glycemic control and may reverse steatosis in patients with T2D and NAFLD/NASH [33]. Interestingly, the primary reason physicians in this study said they prescribed GLP-1 RAs, SGLT2 inhibitors, and pioglitazone was to assist with weight loss; improvement or reversal of steatosis was the second most common reason. Weight loss may be perceived to be a cornerstone of NASH management. While weight loss was the most common reason for prescribing medications that are clinically shown to reduce weight, such as orlistat, SGLT2 inhibitors, and GLP-1 RAs, weight loss was also the most commonly cited reason for prescribing medications that are associated with weight gain (pioglitazone) or are neutral with respect to weight (e.g., DPP4 inhibitors, metformin, and vitamin E). The second most common reason to prescribe the aforementioned medications was to improve/reverse steatosis, an effect for which there is an even greater gap between proven and perceived effect. There is consensus from AASLD and the EASL that metformin is not effective for the treatment of NASH [28, 29]. We found that statins were most commonly prescribed to assist with weight loss and reverse steatosis. While statins are recommended to treat dyslipidemia in patients with NASH [29], even those with cirrhosis [34], statins do not mediate weight reduction. In our study, physicians prescribed statins at similar rates across fibrosis stages. It is possible that these physicians may have been knowledgeable about guidance pertaining to the use of statins in patients with NASH but were, perhaps, disconnected from primary research on why statins are valuable in NASH. Again, further research would be required to confirm this.

### Limitations

This study has several limitations. It does not necessarily reflect diagnosis and treatment patterns in the wider US population, who are managed by providers with less experience than those contributing to the Ipsos NASH Therapy Monitor database. The high rate of evaluating and managing patients with NASH as a specific criterion for physician participation in this study may, for example, explain the high frequency of liver biopsy. An additional limitation is the age of the data in this study. This data was collected in 2016 and 2017, before the most recent AASLD and EASL guidance on NASH and prior to the plethora of data on performance characteristics of non-invasive tests in NAFLD and NASH. If a chart audit was conducted with the same population of physicians today, we may see increased usage of non-invasive tests for the diagnosis of NASH.

## Conclusions

In conclusion, this study shows diagnosis and treatment patterns among a subset of physicians who appear highly engaged with management of NASH. Evaluation and management of NAFLD and NASH in non-academic settings does not, however, discernibly follow established guidelines. There is a need for consensus on the standardization of non-invasive tests for NASH diagnosis. Physicians in this study relied heavily on biopsies for the diagnosis of NASH. In the absence of FDA approved therapies for NASH, much of the pharmacotherapy of NASH is directed at weight loss. Updating and disseminating practice guidelines that are practical and evidence-based may be of high value in attenuating the effects of the NASH epidemic.

## Data Availability

The dataset analyzed during the current study is not publicly available due to the proprietary nature of the Ipsos syndicated NASH Therapy Monitor database. The Ipsos NASH Therapy Monitor is a syndicated patient chart audit owned by Ipsos, an independent global market research company, and data are available to multiple clients on a subscription basis. Data included in this manuscript are © Ipsos 2022, all rights reserved.

## List of Abbreviations

NASH: Nonalcoholic steatohepatitis
NAFLD: Nonalcoholic fatty liver disease
CT: Computed tomography
MRI: Magnetic resonance imaging
PCP: Primary care physician
VCTE: Vibration controlled transient elastography
BMI: Body mass index
SD: Standard deviation
T2D: Type 2 diabetes
CKD: Chronic kidney disease
AST/ALT: Aspartate transaminase/alanine transaminase
AST: Aspartate transaminase
MRE: Magnetic resonance elastography
NFS: NAFLD fibrosis score
ACE: Angiotensin converting enzyme
GLP-1 RA: Glucagon-like peptide-1 receptor agonist
SGLT2: Sodium-glucose co-transporter 2
DPP4: Dipeptidyl peptidase 4
AALSD: American Association for the Study of Liver Diseases
EASL: European Association for the Study of the Liver
US: United States
FDA: Food and Drug Administration
GPP: Good publication practices
